# Broad-Spectrum Antimicrobial Action of Cell-Free Culture Extracts and Volatile Organic Compounds Produced by Endophytic Fungi *Curvularia Eragrostidis*

**DOI:** 10.3389/fmicb.2022.920561

**Published:** 2022-06-23

**Authors:** Hiran K. Santra, Debdulal Banerjee

**Affiliations:** Microbiology and Microbial Biotechnology Laboratory, Department of Botany and Forestry, Vidyasagar University, Midnapore, India

**Keywords:** endophyte, broad-spectrum antimicrobial, volatile metabolite, sustainable agriculture, fungi

## Abstract

Endophytes are the mutualistic microorganisms that reside within the host plant and promote plant growth in adverse conditions. Plants and their endophytes are engaged in a symbiotic relationship that enables endophytes to access bioactive genes of the ethnomedicinal plants, and, as a result, endophytes are constantly addressed in the sector of pharmaceuticals and agriculture for their multidomain bio-utility. The gradual increase of antimicrobial resistance can be effectively countered by the endophytic metabolites. In these circumstances, in the present investigation, endophytic *Curvularia eragrostidis* HelS1 was isolated from an ethnomedicinally valuable plant *Helecteris isora* from East India’s forests. The secondary volatile and non-volatile metabolites are extracted from HelS1 and are found to be effective broad-spectrum antimicrobials. A total of 26 secondary metabolites (9 volatiles and 17 non-volatiles) are extracted from the isolate, which exhibits effective antibacterial [against six Gram-positive and seven Gram-negative pathogens with a minimum inhibitory concentrations (MIC) value ranging from 12.5 to 400 μg ml^–1^] and antifungal (against seven fungal plant pathogens) activity. The secondary metabolite production was optimised by one variable at a time technique coupled with the response surface methodology. The results revealed that there was a 34% increase in antibacterial activity in parameters with 6.87 g L^–1^ of fructose (as a carbon source), 3.79 g L^–1^ of peptone (as a nitrogen source), pH 6.75, and an inoculation period of 191.5 h for fermentation. The volatile metabolite production was also found to be optimum when the medium was supplemented with yeast extract and urea (0.2 g L^–1^) along with dextrose (40 g L^–1^). Amongst extracted volatile metabolites, 1-H-indene 1 methanol acetate, tetroquinone, *N*, *N*-diphenyl-2-nitro-thio benzamide, *Trans* 1, 2-diethyl-*trans*-2-decalinol, naphthalene, and azulene are found to be the most effective. Our investigation opens up opportunities in the sector of sustainable agriculture as well as the discovery of novel antimicrobials against dreadful phyto and human pathogens.

## Introduction

Natural products, such as antibiotics, anti-fungal, anti-cancer, and other pharmaceutically valuable agents from plants and microbial sources, have always been the front warriors against dreadful infectious and non-infectious diseases (Tyrrell et al., 2006). The indiscriminate and irrational use of synthetic chemotherapeutic agents has led to intense and deleterious consequences of antimicrobial resistance (AMR) and the rapid development of multi-drug resistant strains (MRSA, methicillin-resistant *Staphylococcus aureus*; VRSA, vancomycin-resistant *S*. *aureus*; and PRSA, penicillin resistant *S*. *aureus*, etc.) (Deshmukh et al., 2022). Presently, the popular classes of antibiotics are losing their efficacy. The emergence of new and reemergence of old infectious diseases has made the situation difficult to combat those pathogens (Manganyi et al., 2019; Wang et al., 2019). The rising cases of AMR are roughly estimated to take the lives of 10 million people globally by 2050, and there might be an economic loss of a total of 100 trillion US$, affecting a number of 24 million global citizens (Sugden et al., 2016).

Fungal pathogens are a serious threat to domestic and commercial agrarian systems and largely affect the global food supply and economy. Fungal pathogens are reported to cause 70–80% of livestock and post-harvest infections in fruits, vegetables, cereals, and pulses, which ultimately affect the global demand-and-supply chain (Santra and Banerjee, 2020a). The increasing human population demands a simultaneous increase of 60–110% of the global food pool by 2050 to equilibrate the production-consumption ratio. But croplands are losing their fertility and productivity due to overuse of chemical fertilizers and fungal attacks, respectively (Edgerton, 2009; Liu-Xu et al., 2022). The use of chemical pesticides and systemic fungicides is the most popularly adopted solutions to tackle this problem but leads to detrimental side effects concerning human health due to the incorporation of xenobiotics in the food chain and the development of resistant micro-organisms. The situation demands the natural products derived from plants and microbes, which may be an alternative way to maintain sustainable ecosystem practices (Llorens et al., 2019). VOCs derived from endophytes are a very potent myco-fumigator and gaining interest in the agro-research industry (Saxena and Strobel, 2021).

Now, it is high time to switch on the search for novel anti-microbial compounds from biological entities; in recent times, endophytic microorganisms have had the most potent candidate in this field (Hussein et al., 2022; Maliehe et al., 2022; Sharma et al., 2022). Endophytes are hidden microbial entities that reside within the host plant and promote the growth of the plant under detrimental conditions and also possess immense pharmaceutical and biotechnological importance (Charria-Girón et al., 2021; Wolfe et al., 2022). They represent a wide range of microorganisms (bacteria, actinobacteria, fungi, mycoplasma) and are ubiquitous in their occurrence (Mishra et al., 2017, 2022; Yuan et al., 2022). It has been reported that plants harbor a wide spectrum of bioactive secondary metabolites (tannin, terpene, organic acids, aromatic volatiles, phenols, polysaccharides) for their existence. Endophytes are co-evolved partners with their host plants through horizontal gene transfer and can synthesize bioactive compounds similar to the host plant metabolites. The endophytic metabolites are pharmaceutically and agriculturally valuable and can be utilised for the development of antimicrobial products.

Our present investigational outcome has addressed the antimicrobial resistance and crop loss problems using endophytic secondary metabolites as a bio-weapon. In this study, an ethnomedicinally valuable plant *H*. *isora* from East Indian Forest is selected for an endophyte-related study, and an endophytic *Curvularia eragrostidis* HelS1 has been isolated from the leaf tissues of the host plant. *H*. *isora* as a whole or its different parts like stem-bark, leaves, fruits, seeds, and roots possess potent antimicrobial, anti-oxidant, hepato-protective, anti-plasmodial, anti-cancer, anti-nociceptive action and are randomly used as Ayurvedic and Unani medicines (Srinivasa et al., 2022; Tiwari and Bae, 2022). The secondary metabolites extracted from HelS1 endophyte are found to be effective anti-bacterial agents restricting the growth of pathogenic MRSA, and *Vibrio parahaemolyticus*, causing lethal leakage of necessary macromolecules, blocking bio-film formation, and hampering the central carbohydrate metabolism.

In this regard, the present study has been focused on the isolation, extraction, and testing of the antibacterial efficacy of endophytic fungi, which can lead to the discovery of new antimicrobial agents in controlling post-harvest decay/loss of standing crops and clinical infections, respectively.

## Materials and Methods

### Collection of Plant Materials

Disease-free, healthy, and mature leaves of *Helicteres isora* (also called as an East India screw tree) were collected from forests near Burudhi lake areas of Ghatshila (22° 36′ 2′′ N, 86° 26′ 47′′ E), East Singhbhum district, Jharkhand, India, and brought to the Microbiology and Microbial Biotechnology Laboratory, Vidyasagar University in an icebox (Tarson, India) for further study endophytic fungal flora.

### Isolation and Characterisation of Endophytic Fungi

Leaves were surface sterilised first in running tap water and then in sodium hypochlorite (4%)-NaOCl (HiMedia, India), H_2_O_2_ (3%)-hydrogen peroxide (HiMedia, India) solution for the removal of surface microorganisms (Schulz et al., 1993). Leaves were cut into small pieces (0.5 cm × 0.5 cm) by sterile blades and were flamed in a spirit burner for surface drying after emerging in 70% alcohol for 5–10 s. In total, 150 explants were placed on water agar Petri plates (Borosil, India) and incubated in a BOD incubator (SN Solutions, India) at 30°C for 3 to 5 days. The effectiveness of this sterilisation and isolation process was cross-checked by the explant imprintation technique described by Schulz et al. (1993). After 48 to 72 h of incubation, the endophytic fungal hyphae emerge from explant tissues. Agar blocks with fungal hyphae were transferred to a Potato Dextrose Agar (PDA; HiMedia, India) medium for further identification studies. Approximately, 150-μg ml^–1^ of streptomycin was mixed with agar plates to avoid bacterial contamination. Isolates were characterised based on their morphology using light (Primo Star, Zeiss, Germany) and a stereo-microscope (Stemi 508, Zeiss, Germany). The colonisation frequency of each isolate, along with diversity indices like Shannon–Wiener, Simpson’s dominance, Simpson’s diversity, and species richness, was also calculated, following standard methods (Simpson, 1951; Hata and Futai, 1995; Gond et al., 2012).

### Molecular Identification of the Most Potent Isolate

Endophytic fungi were subjected to rDNA-based molecular identification according to the standard protocols (Mahapatra and Banerjee, 2012). Partial sequencing of ITS1, a large subunit of ribosomal RNA gene along with a complete sequence of 5.8S ribosomal RNA and ITS2, was done. Further analysis was done using the 552-base pair of the consensus sequence. Sequences were submitted to GenBank. The sequences obtained in this study were compared to the GenBank database using BLAST. Fourteen sequences, including HelS1, were selected and aligned using the multiple alignment software program Clustal W, and the phylogenetic tree was constructed using MEGA 10 (Tamura et al., 2007).

### Koch’s Postulation Test for Confirmation of the Endophytic Nature of the Selected Isolate

Several *H*. *isora* plants were selected and transplanted to the Vidyasagar University greenhouse facility. Leaves were pinpricked two to three times in each of many leaves, and the leaf surfaces were inoculated with a fungal suspension of 10^8^ spores ml^–1^. Another set of uninoculated leaves was treated in the same manner but without the introduction of the endophytic fungal spore suspension. The plants were placed at 23°C in 100% relative humidity for 7–9 days and then checked for the occurrence of symptoms. Re-isolation of the putative endophyte was done in the same manner (from the surface-sterilised leaves) as described earlier for endophytic fungal isolation, and reisolated fungi were identified based on cultural and morphological characters (Strobel et al., 2011).

### Antibacterial Activity of Cell-Free Supernatant of Endophytic Fungi

#### Screening of Endophytic Fungal Isolates for Production of Antibacterial Compounds

To detect the potent endophytic fungi with antibacterial activity, isolates were grown in 100 ml of PDB (Potato Dextrose Broth, HiMedia, India) for 5–8 days at 28°C, with a mild agitation of 125 rpm in dark conditions. Periodically, at an interval of 2 days, 10 ml of fungal culture broth was withdrawn, and cell-free supernatant (CFS) was prepared by filtering it using a muslin cloth, followed by centrifugation (10,000 rpm for 10 min) in a Remi R24 centrifuge, India. CFS was assessed for antibacterial activity by a disk diffusion technique against a wide range of both Gram-positive; *B*. *cereus* (ATCC, 14579), *B*. *subtilis* (ATCC, 11774), *Staphylococcus aureus* (ATCC, 29213), MRSA (ATCC, 33591), *Staphylococcus epidermidis* (MTCC, 2639), *Listeria monocytogenes* (MTCC, 657), and Gram-negative; *Shigella flexneri* (MTCC, 1457), *Shigella dysenteriae* (ATCC, 13313), *Klebsiella pneumoniae* (ATCC, 75388), *P*. *mirabilis* (ATCC, 12453), *P*. *aeruginosa* (ATCC, 9027), *V*. *parahaemolyticus* (ATCC, 17802), *E*. coli (MTCC, 4296) pathogenic microorganisms. Approximately, 5-mm diameter sterile disks were soaked with 50 μL of culture filtrate and were placed at the MHA (Mueller Hinton Agar, HiMedia, India) plates inoculated with test microorganisms and incubated at 37°C. After 24 h, zones of inhibition (in mm) were observed, and diameters were measured. Streptomycin, clindamycin, vancomycin, and ciprofloxacin (HiMedia) were used as standard antibiotics.

#### Physico-Chemical Characterization of Antibacterial Compounds

The cell-free supernatant (CFS) of the most potent antibacterial producer HelS1 was kept in a boiling water bath for 10 min to check its thermostability. Furthermore, to detect the proteinaceous nature of the antibacterial components, CFS- was treated with proteinase K (Thermo Scientific, United States) at a concentration of 1 mg ml^–1^ for 2 h at 37°C. The antibacterial activity of both heat-killed and proteinase-K-treated cell-free supernatant was studied against the pathogenic bacteria by the disk diffusion method with appropriate control (Fernandez-Garayzabal et al., 1992).

#### Extraction of Endophytic Fungal Metabolites

Based on the maximum antibacterial production, PDB was selected as the most suitable medium for fungal culture and solvent extraction. HelS1 was inoculated in 1 L of PDB in a 2-L Erlenmeyer flask at 28°C for 12 days, and CFS was obtained by filtering the broth with muslin cloth, followed by centrifugation (10,000 rpm for 15 min). For every 100 ml of the CFS, 200 ml of ethyl acetate (EA, HiMedia) was added and shaken vigorously for 30 min using a magnetic stirrer, and the EA layer was separated using a separating funnel. A Vacuum Rotary Evaporator (Bacca Buchi, Switzerland) was used to evaporate the excess EA. The remnants were suspended in DMSO (dimethyl sulphoxide, HiMedia, India) and stored for further bioactivity studies.

#### Determination of Minimum Inhibitory Concentrations and Minimum Bactericidal Concentration

Antibacterial activities of EA fraction of HelS1 CFS were determined against both Gram-positive and Gram-negative bacterial pathogens by a disk diffusion technique as described earlier (Bauer, 1966). EA fractions were dissolved in DMSO in different concentrations, and 15.62 μg ml^–1^ to 4 mg ml^–1^ of culture extract was prepared according to the broth micro-dilution method for the determination of MIC and minimum bactericidal concentrations (MBC) values in a 96-well round-bottom microtitre plate (Merck, Germany), following the guidelines of Clinical and Laboratory Standards International (CLSI). In the case of the MIC assay, 100 μL of fractions with varying concentrations (0.195 μg ml^–1^ to 3.2 mg ml^–1^) was added to 100 μL of MHB (1.5 × 10^8^ CFU ml^–1^ prepared according to the 0.5 McFarland turbidity standard) in wells. Antibiotic ciprofloxacin and MHB (containing bacteria only) were selected as positive control and negative control, respectively. The plate was incubated at 37°C for 24 h. Quantification of the optical density in each well was carried out using a microplate reader (ROBONIK readwell Touch ELISA Plate Analyser, India). Wells showing zero growth on the plate were referred to as MBC.

#### Preparation of a Time-Kill Curve

The killing kinetics experiment was performed following the standard guidelines (Lorian, 2005). Fresh bacterial cultures of four pathogenic microorganisms; – MRSA, *S*. *epidermidis*, *V*. *parahaemolyticus*, and *S*. *flexneri* were prepared separately in MHB and were suspended in sterile saline water. The culture turbidity was adjusted at 0.5 McFarland’s standard for inoculation. The suspension was diluted further to obtain an inoculum of 5 × 10^7^ CFU ml^–1^. DMSO that dissolved EA extract (HelS1 CFS) was prepared in different concentrations (MIC/2-half of MIC, MIC, MIC×2-double of MIC, MIC×4-four times of MIC) and was added to different flasks, containing a bacterial suspension of MRSA, *S*. *epidermidis*, *V*. *parahaemolyticus*, *S*. *flexneri* separately. In total, five sets of bacterial samples along with different concentrations of fungal EA were prepared for each bacterium. No addition of the fungal extract to a flask indicates the negative control. Bacterial cultures were harvested at different time intervals from the experimental setup and in total five-time points (0, 2, 4, 6, and 24 h) were selected for sampling, following centrifugation at 37°C at 110 rpm. The samples were serially diluted and re-cultured in the Muller Hinton agar and then incubated overnight at 37°C. To determine log CFU values, the plotted graph was defined as bactericidal or bacteriostatic based on the CFU reduction at various time intervals. The compounds were said to be bactericidal if they exhibited ≥ 3 log CFU (a colony forming unit) reduction and bacteriostatic if they exhibited <3 log CFU reduction.

#### Estimation of the Release of Intracellular Components From Pathogenic Cells

To check the effects of EA fraction of HelS1 on bacterial cell membrane permeability or cellular integrity, the release of intracellular macromolecules, i.e., protein, nucleic acid, and K+ ions on the extracellular environment, was checked. Gram-positive bacteria MRSA, *L*. *monocytogenes*, and Gram-negative bacteria *Pseudomonas aeruginosa* and *V. parahaemolyticus* were grown (OD620 nm = 0.5) in 250-ml MH broth, and bacterial cells were harvested by centrifuging the culture broth for 10 min at 6,000 rpm. The cell pellets were taken and washed two times with a 50-mM Na-P buffer (pH, 7.). The cell pellets were resuspended in 1 ml of the same 50-mM Na-P buffer. Then, the pellets were treated with EA extract of HelS1 at its MIC and MBC values. After 6 and 24 h of incubation, the complete solution was centrifuged at 10,000 rpm for 10 min to obtain the cell-free extracts. Protein and DNA concentrations in the mixture were determined following the methods of Lowry et al. (1951) and Burton (1956), respectively, using a Shimadzu UV 1800 spectrophotometer (Japan). K^+^ ions concentration was calculated using a flame photometer (Elico, CL-378, India), with K_2_HPO_4_ (Merck, Germany) as a standard. The set that was only treated with DMSO was considered as a control.

#### Study of the Effect of Ethyl Acetate Fraction on Bacterial Key Enzymes

Four different Gram-positive and Gram-negative bacterial pathogens, MRSA, *L*. *monocytogenes*, *V*. *parahaemolyticus*, and *Pseudomonas aeruginosa*, were treated with the EA fraction of HelS1, respectively (MIC and MBC) for 24 h. The cells were harvested by centrifugation at 6,000 rpm for 10 min. Then, the harvested cells were washed with Na-P buffer solution (20 mM) and again suspended in 1 ml of the same buffer. After that, the cells were ruptured (using sonicator- Labsonic M) to obtain the cell-free extracts. Finally, the supernatants were collected by centrifugation at 10,000 rpm for 10 min and used as a crude enzyme. The most three physiologically valuable enzymes, FBPase (fructose-1,6-bisphosphatase), PFK (phosphofructokinase), and ICDH (isocitrate dehydrogenase), were assayed following the methods of Mandal and Chakrabarty (1993). Other necessary components, such as substrate, enzymes (cell-free extracts of individual bacterial pathogens), and co-factor, were mixed in a cuvette. The rate of NADP reduction was followed at 340 nm in a UV-Vis spectrophotometer (Shimadzu UV-1800). The specific activity was calculated as nanomoles of substrate consumed per minute per mole protein and compared to the untreated control.

#### Study of Inhibition of Biofilm Formation

Biofilm formation of the bacterial pathogens was tested using a 24-well polystyrene cell culture plate. One milliliter of MH broth was poured into each well and then inoculated with a 1% fresh culture of bacterial pathogens. The EA fraction of HelS1 (dissolved in 10% sterilised DMSO) was added at different concentrations to different wells. After an incubation of 48 h at 27°C, the broth was decanted from every well and then washed with sterilised distilled water without hampering the biofilm formation. After that, each well was dried and then washed gently with sterilised water without disturbing the biofilm. Next, the wells were stained with 1 ml of 0.1% crystal violet (SRL) and kept at room temperature for 10 min. The crystal violet stain was washed, and 1 ml of 33% acetic acid (SRL) was added to each well and then kept for 30 min at room temperature, providing mild agitation to extract the bound crystal violet from bacterial cells. The optical densities (OD) of acetic acid solution were then measured at 595 nm using a UV-vis spectrophotometer. The inhibition of the biofilm was determined according to the formula [60]% inhibition of biofilm formation = 100 – [(OD570 of sample/OD570 of control) * 100]. The biofilm formation was classified at three levels: the highest one (OD570 ≥ 1), intermediate (0.1 ≤ OD570 < 1), and no formation of biofilm (OD570 < 0.1).

#### Synergistic Activity of Ethyl Acetate Fraction of HelS1 and Antibiotic Ciprofloxacin

The synergistic antibacterial activity of the HelS1 EA fraction and broad-spectrum antibiotic ciprofloxacin against MRSA were tested, following the checkerboard method proposed by Orhan et al. (2005) with slight modifications. The EA fraction of the endophytic fungi HelS1 (0–1,000 μg ml^–1^) and the most effective antibiotic ciprofloxacin (0–100 μg ml^–1^) were used at variable combinations. A total of 1 ml of MH broth was taken to a 1.5-ml microcentrifuge tube and then inoculated with 1% MRSA culture (OD620 nm = 0.5). HelS1 EA extract and ciprofloxacin were mixed at variable combinations. The mixture was incubated at 28°C for 48 h. The cell pellets were drawn after centrifugation for 10 min at 6,000 rpm and washed thoroughly two times in a 50-mM Na-P buffer. Next, the bacterial cells were suspended in 1 ml of the same buffer, and the OD values were measured at 620 nm to quantify the cellular amounts. To calculate the ΣFIC (fractional inhibitory concentration), different OD values were placed on the checkerboard. ΣFIC was calculated by the following formula, ΣFIC = FIC of EA fraction + FIC of ciprofloxacin, where the FIC of the EA fraction or ciprofloxacin = MIC of the EA fraction or ciprofloxacin in combination/MIC of the EA fraction or ciprofloxacin alone. The combination of endophytic culture extract and ciprofloxacin was considered antagonistic when ΣFIC >4, synergistic when ≤0.5, and indifferent when >0.5 (Prinsloo et al., 2008).

#### Optimisation of Culture Conditions for the Production of Maximum Antibacterial Metabolites

The valuable growth parameters like incubation temperature, incubation time, medium p^H^, additional carbon, nitrogen, and salt sources, and their most suitable concentration for optimum growth are measured using the OVAT (one variable at a time) system. Furthermore, the RSM (response surface methodology) technique was adopted by BBD (Box Behnken Design) to find out the most valid statistical model for maximum production of antibacterial components (Mahapatra and Banerjee, 2013).

### The Anti-fungal Potential of Endophytic Fungi

#### Anti-fungal Action of Endophytic Fungal Volatile Organic Compounds

The isolates were further checked for their antifungal VOCs (volatile organic compounds) production against selected pathogens; *Rhizoctonia solani* (MTCC-4634), *Alternaria alternata* (MTCC-3793), *Fusarium oxysporum* (MTCC-284), *Geotrichum candidum* (MTCC-3993), *Botrytis cinerea* (MTCC-8659), *Cercospora beticola* (ATCC-12825), *Aspergillus fumigatus* (MTCC-3785), *Ceratocystis ulmi* (ATCC-32437), *Pythium ultimum* (ATCC-200006), following a bioassay method proposed by Strobel et al. (2001). Antifungal VOC production was checked at different time intervals (after 4th, 5th, 6th, and 7th days). A 1-cm wide strip of agar was cut off from the center of a standard Petri plate (100 mm × 15 mm) of PDA, forming two halves of agar. Endophytic fungi were inoculated onto one side of the plate (half-moon agar) and incubated at 24°C for 4–8 days for maximum production of bioactive VOCs. Test pathogenic fungi were inoculated on the other half-moon agar side of the Petri plate from the 4th until the 8th day. Petri plates were then wrapped for two-three times with parafilm and incubated at 24°C for 48–96 h. The growth of filamentous fungi was quantitatively assessed based on multiple measurement criteria of growth relative to control. Tests were conducted in triplicate.

### Calculation of IC_50_ Values of an Artificial Mixture of Volatile Organic Compounds

The artificial mixture of authenticated VOCs was made by mixing the standards of volatiles purchased from Sigma in a particular ratio (following the relative ratio of VOCs emitted from endophytic *Curvularia eragrostidis* HelS1). The artificial mixture of VOCs was poured into a pre-sterilised micro cup (4–6 mm) and placed in the center of a PDA Petri plate. Agar blocks (5 mm × 5 mm × 5 mm) with pathogenic fungal hyphae were placed on the Petri plate at a 2.5-cm distance from the VOCs containing a micro cup, and they were wrapped with two layers of Parafilm. Mycelial growths were measured after 48 h of incubation. Control plates lack artificial mixtures. Tests on 2–40 μL of the artificial mixture per 50 ml of air space above the mycelial culture in the PDA plate were performed on four replicates, and IC50 values were calculated.

### Optimization of Culture Media for Maximised Volatile Organic Compounds Emission

Endophytic fungi were grown on basal media with alterations in carbon and nitrogen sources to optimize the maximum production of VOCs. At first, a 5-mm fungal block was transferred to different media compositions like different nitrogen sources-beef extract, tryptone, peptone, yeast extract, and different carbon sources-cellulose, dextrose, and malt extract. The emission of naphthalene (the highest occurrence amongst other volatiles) was assessed qualitatively due to its moth ball-like odor. A rating of 0–5 has been assigned to each media composition based on olfactory observations of VOCs made by five different individuals [23]. The media compositions that were used for the VOC emission assay are as follows; -**1**- Beef extract, 0.2 g L^–1^ with salts; **2**- Tryptone, 0.2 g L^–1^ with salts; **3**- Peptone, 0.2 g L^–1^ with salts; **4**- Yeast extract, 0.2 g L^–1^ with salts; **5**- Yeast extract, 0.2 g L^–1^ with salts plus cellulose, 40 g L^–1^; **6**- Tryptone with salts plus cellulose, 40 g L^–1^; **7**- Yeast extract, 0.2 g L^–1^ with salts plus dextrose, 40 g L^–1^; **8**- Tryptone, 0.2 g L^–1^ plus dextrose, 40 g L^–1^; **9**- Yeast extract, 0.2 g L^–1^ plus malt extract, 40 g L^–1^; **10**- Tryptone, 0.2 g L^–1^ plus malt extract, 40 g L^–1^; **11**- Potato Dextrose Agar (PDA) (Hi-Media); **12**- Potato Carrot Agar (PCA) (Hi-Media); **13**- Oatmeal Agar (OA) (Hi-Media); **14**- instant smashed potato dextrose agar (potato, 4 g L^–1^, dextrose-20 g L^–1^); **15**- modified PDA (instantly smashed potato, 4 g L^–1^; yeast extract and urea, 0.2 g L^–1^; dextrose, 40 g L^–1^); **16**- modified CDA (yeast extract and urea, 0.2 g L^–1^; dextrose, 40 g L^–1^); **17**- modified oatmeal (yeast extract and urea, 0.2 g L^–1^; dextrose, 40 g L^–1^). In each case, agar and salt concentrations were maintained according to the modified M1-D medium (Pinkerton and Strobel, 1976). All the materials used for media preparation were purchased from SRL and HiMedia.

### Identification of the Bioactive Compounds

#### Thin Layer Chromatographic Analysis of Ethyl Acetate Fraction

Partial purification of the bioactive metabolites of the HelS1 extract was performed using thin-layer chromatographic techniques. The dried fungal extract was resuspended in EA, maintaining a concentration of 20 mg ml^–1^, and 10 μL of EA extract was loaded onto alumina–silica Thin Layer Chromatographic (TLC) plates (MERCK Silica gel F254) using capillary glass tubes. Acetone (HiMedia) and n-hexane (HiMedia) in a ratio of 2:8 were used as running solvent, and the retention factor was calculated for all the bands under UV light using a Camag UV Cabinet. The bioactive compounds corresponding to each band were scratched and collected and were finally dissolved in 1 ml of EA, which was centrifuged (7,000 rpm for 15 min) and evaporated to dryness. The dried components were then dissolved in DMSO at 100 μg ml^–1^ concentration, and antibacterial action was measured by disk diffusion technique (Wang et al., 2014).

#### Gas Chromatography-Mass Spectrometry Analysis of the Antibacterial and Antifungal Metabolites

The active fractions obtained from TLC analysis were analysed using Gas Chromatography (TRACE 1300)-Mass (ISQ QD Single Quadrupole) Spectrometry (GC-MS) system- Thermo Scientific (USA, Waltham, MA, United States) with an ESI mode. The instrument was configured with a DB-5 Ultra Inert column (30-m length and a 0.25 mm inner diameter) for a 22-min run of a 1 μL sample (a split-less flow) with an injector port and oven temperature of 240 and 50°C, respectively, having 10°C min^–1^ ramping time up to 260°C with helium as the carrier gas. Tri-plus RSH-based automated injection was done. The flow velocity of the carrier gas was set at 1 ml min^–1^. The ionisation source was kept at 250°C with 70 eV of ionisation energy and 0.1 kV ionisation current. The mass fragmentation pattern was analysed by X-Calibur software. The identification of the various compounds was based on the SI and RSI value with the best-matched compound in the NIST library (Tomsheck et al., 2010).

Qualitative analyses of the volatiles emitted by the fungal endophyte were done using the Solid Phase Microextraction (SPME) fibre technique (Mitchell et al., 2009). The endophytic fungus was grown on a 50 ml gas vial (Thermo Scientific, United States), containing 5 ml of modified PDA (described previously) media, forming a slant and was incubated for 8 days at a temperature of 24°C. At first, the inoculated GC-Glass vial was heated at 40°C for 45 min in the incubation cabinet attached to the Tri-Plus RSH autosampler unit of the GC-MS instrument. The incubation cabinet was provided with mild agitation in clockwise and anti-clockwise orientations (five agitations per minute). Then, a baked SPME syringe (Supelco), consisting of 50/30 divinylbenzene/carburen on polydimethylsiloxane on a stable flex fibre, was injected into the GC-Gas vials through the magnet-based rubber cap and was exposed to the vapour phase for 45 min. The SPME fibre was then injected into the splitless injection port of the system, containing the ZB-Wax column (a 30 m diameter, a 0.25 mm inner diameter, 0.50 mm thickness). The column was set at 30°C for a 2-min hold, followed by an increase to 225°C at 5°C min^–1^. Helium was used as the carrier gas with a flow velocity of 1 ml min^–1^. Additionally, the column head pressure was maintained at 50 kPa. The SPME syringe was conditioned at a temperature of 240°C for 25 min under a flow of helium gas (1 ml min^–1^). A 30 s injection time was used to introduce the sample fibre into the system. Bioactive components were identified following the NIST library databases and listed in the NIST terminology. The authenticity of the compounds was made according to the GC/MS of authentic standards (Sigma–Aldrich). Compounds that were not identified using the NIST library match were identified as the NIST library database only when they provide a quality score of 60 or better. Experiments were repeated two times.

### Statistical Analysis

All experiments were performed in triplicate, and the results are presented as means ± standard errors (SE). Data were analysed by Prism GraphPad software version 9.2.0 (332) (San Diego, CA, United States). Minitab (version 20.2) statistical software was used for response surface methodology experiments (Box–Behnken design).

## Results

### Identification of the Isolates and Selection of the Potent Endophytic Fungi

In total, eight endophytic fungi were isolated from the leaf samples of the *Helicteres isora* plant. The non-epiphytic nature of the fungal isolates was confirmed by the tissue fingerprinting method. Briefly, the aliquots with wash liquid of the explants were plated on PDA, and there was no occurrence of any endophytic fungi. The endophytes were non-pathogenic as they were isolated from healthy disease-free plants. Further confirmation was provided by Koch’s Postulate results. There were no disease symptoms developed after inoculation of the endophytic fungi onto the *H*. *isora* host plant.

In total, 107 endophytic fungal taxa were obtained from the 150 samples (explants) of leaf tissues. Eight types of fungi were found, and isolates were identified as two species of Fusarium, one species of Pestalotiopsis, one species of Penicillium, two species of Aspergillus, and two different species of sterile isolates (HelS1 and HelS2). The two sterile isolates exhibited the highest colonisation frequency of 14 and 10.66%, respectively. The lowest colonisation frequency (4.66 and 6%) on the leaf tissues was shown by the two species of Aspergillus (Supplementary Table 1, Supplementary Figure 1). The diversity of the endophytic community of leaf tissues is represented in tabular form (Supplementary Table 2). The results of diversity indices indicate an acceptable range of endophytic fungal diversity in the leaf tissues of the explant.

**FIGURE 1:**
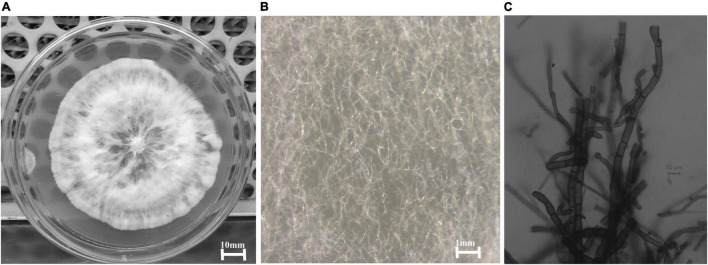
**(A)** A 6-day old culture of *Curvularia eragrostidis* HelS1 grown on PDA. **(B)** Sterile mycelial hyphae of the isolate seen under a stereo-microscope. **(C)** Septate sterile hyphae seen under a light microscope.

The two isolates did not produce any reproductive structures and remained sterile even after inoculation in a leaf carnation agar medium and remained as unidentified. The isolates were tested for their antibacterial action, and one unidentified (isolate code-HelS1) isolate exhibited the highest antibacterial activity. The sterile isolate (HelS1) was identified as *Curvularia eragrostidis* HelS1 (Gen Bank Acc. No.-ON146362). Plate morphology, along with microscopic morphology, is recorded in Figure 1.

**FIGURE 2:**
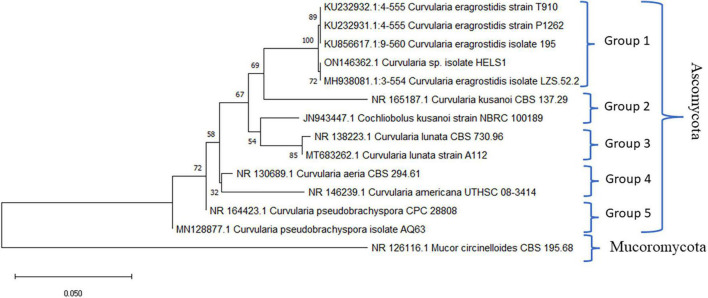
A phylogenetic tree of the endophytic isolate *C*. *eragrostidis* HelS1.

Molecular identification involving two primers ITS1 and ITS4, aiming at the conserved regions 28S rDNA and 18S rDNA, respectively, was utilised for the amplification of the ITS1-5.8S-ITS2 region of the endophytic fungal isolate HelS1. The gene products obtained through amplification were further sequenced, and derived results were assured by comparing the sequences against the available Genbank database of NCBI using the BLAST tool. Gaps and missing data were removed from the dataset. There were a total of 552 nucleotides in the final dataset. A phylogenetic tree was constructed to compare the sequence of HelS1 with the existing fungal nucleotide database in Genbank. Evolutionary distances were analysed by using the neighbour-joining method, and bootstrap analysis was performed to construct the phylogenetic tree, which is represented in Figure 2 (Saitou and Nei, 1987; Tamura et al., 2004). The tree depicts that the endophytic fungal isolate is phylogenetically related to the *Curvularia eragrostidis*, and it is supported by high bootstrap values (72%). Five hundred bootstrapping repetitions were performed for the construction of cladograms. The phylogram constituted was divided into five groups (Felsenstein, 1985). The differentiation of the tree into five groups was based on different species of Curvularia utilised to set up the tree with a high-to-low similarity index. Different strains of five different species of Curvularia (Division-Ascomycota) were involved in constructing the phylogenetic tree, namely, *Curvularia eragrostidis*, *C*. *kusanoi*, *C*. *lunata*, *C*. *aeria*, *C*. *americana*, *C*. *pseudobrachyspora*. Another member of Mucoromycota-*Mucor* sp. was taken as the outgroup. The tree starts with *C*. *eragrostidis* strain T910 along with strains P1262, 195, and LZS52.2 and includes our endophytic isolate HelS1. All these strains are clustered into Group 1, with a high bootstrap value (72%). Groups 2 and 3 contain two species of *C*. *kusanoi* and *C*. *lunata*, respectively, whereas *C*. *aeria*, *C*. *americana*, and *C*. *pseudobrachyspora* constitute Groups 4 and 5, respectively. The total branch length of the phylogenetic tree was calculated as 0.73840605. The bioactive endophytic fungal isolate HelS1 evaluated in this study completely matches (a 100% query cover) with *C*. *eragrostidis* LZS 52.2 and, hence, can be concluded as *C*. *eragrostidis* HelS1.

### Thermostable and Non-proteinaceous Nature of the Metabolites of HelS1

Heat-killed and proteinase K-treated extracts of HelS1 exhibited antibacterial action (in terms of a clear zone of inhibition in mm) same as the untreated one. The control sets containing uninoculated broth and proteinase K (1 mg ml^–1^) did not exhibit any clear zone of inhibition.

### Antibacterial Activity of HelS1 Metabolites

Isolate HelS1 exhibited the maximum antibacterial action and was selected for further antibacterial evaluation. PDB was selected as the most suitable medium for antibacterial production, and EA was selected as the most potent solvent for extraction of antibacterial metabolites. The EA extract of HelS1 exhibited broad-spectrum antibacterial action against 13 Gram-positive (6) and Gram-negative (7) pathogens. MIC and MBC values of EA extract against all the pathogenic microorganisms ranged from 12.5 to 100 μg ml^–1^ and 200 to 400 μg ml^–1^ for Gram-positive and Gram-negative pathogens, respectively (Table 1). The EA extract is found to be heavily effective against Gram-positive pathogens than the Gram-negative ones.

**TABLE 1:** Antibacterial activity-minimum inhibitory concentrations (MIC-μg ml^–1^) and minimum bactericidal concentrations (MBC-μg ml^–1^) of EA (ethyl-acetate) culture extract of HelS1 against pathogenic bacteria.

Pathogenic bacteria	MIC (μgmL^–1^)	MBC (μgmL^–1^)
**Gram-positive pathogens**		
*B*. *cereus* (ATCC-14579)	12.5	25
*B*. *subtilis* (ATCC-11774)	12.5	25
*S*. *aureus* (ATCC-29213)	50	100
Methicillin-resistant *Staphylococcus aureus* (MRSA; ATCC 33591)	100	200
*S*. *epidermidis* (MTCC 2639)	25	50
*L*. *monocytogenes* (MTCC657)	100	200
**Gram-negative pathogens**		
*S*. *flexneri* (MTCC 1457)	200	400
*S*. *dysenteriae* (ATCC 13313)	400	800
*V*. *parahaemolyticus* (ATCC 17802)	400	800
*K*. *pneumoniae* (ATCC 75388)	200	400
*P*. *mirabilis* (ATCC 12453)	400	800
*P*. *aeruginosa* (ATCC 9027)	200	400
*E*. *coli* (MTCC 4296)	400	800

### Time-Killing Kinetics of Bacterial Pathogens Upon Treatment With HelS1 Metabolites

**FIGURE 3:**
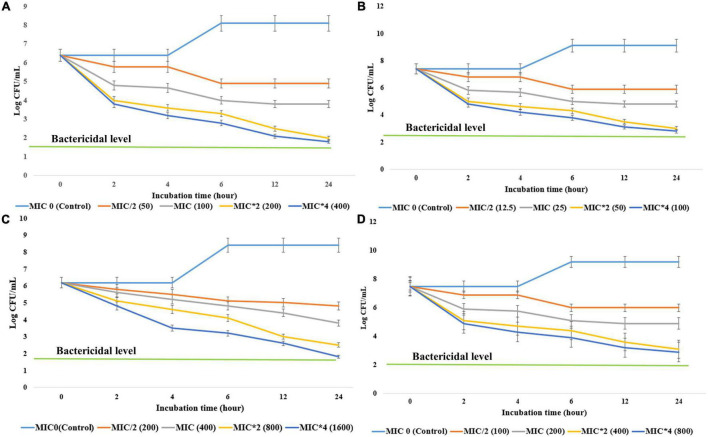
The effect of different concentrations (minimum inhibitory concentrations: MIC/2, MIC, MIC*2, MIC*4) of antibacterial compounds of HelS1 on the growth of bacterial pathogens [**(A)** methicillin-resistant *Staphylococcus aureus* (MRSA), **(B)** *S*. *epidermidis*, **(C)** *S*. *dysenteriae*, **(D)** *K*. *pneumonia*] is represented here in the form of a time-kill curve. The CFUs (colony-forming units) are counted after 0, 2, 4, 6, 8, 10, 12, and 24 h of treatment. Values on the graphs are the means ± standard error (SE) of the three replicates.

To determine the bacteriostatic or bactericidal nature of antimicrobial components, EA fraction of HelS1 was added into the active mid-log phase culture of MRSA, *S*. *epidermidis*, *S*. *dysenteriae*, and *K*. *pneumoniae* at different concentrations- MIC *O (control), MIC/2 (half of MIC), MIC, MIC*2 (double of MIC), MIC*4 (fourth times of MIC). The control set, which has not received any EA extract treatment, is considered the MIC*0. CFU counting at an interval of 2 h revealed that there was a rapid decline in microbial growth as a result of treatment with the fungal extract. The antimicrobial compounds exhibited 3 log CFU reduction at 100, 25, 400, and 200 μg ml^–1^, respectively, against MRSA, *S*. *epidermidis*, *S*. *dysenteriae*, and *K*. *pneumoniae* after 12–18 h of treatment in all the cases. The <3 log CFU reduction denotes the MBC of the bioactive compounds. Detailed growth kinetics is represented in Figure 3.

### Leakage of Intracellular Essential Molecules

Intracellular macromolecules like DNA, protein, and K^+^ ions were found to be leaked in the extracellular environment after treatment with HelS1 EA extract, which specifies the cidal action of endophyte-derived bioactive compounds. The cell bursting effect was more massive in Gram-positive pathogens than in Gram-negative ones. There was a 1.5- to-2-fold increase (*p* < .001) of DNA, protein, and K^+^ ion contents after 24 h of treatment in comparison to 6-h treatment (Supplementary Figure 2). The presence of K^+^ ions on the extracellular fluid indicates that the bacterial cell integrity is disturbed upon treatment.

### Biofilm Inhibition Potential of HelS1 Metabolites

UV-VIS spectrophotometric measurement of a biofilm after crystal violet staining revealed an inhibition (*p* < 0.001) of biofilm development of pathogenic strains as a result of treatment with HelS1 EA fraction. The pathogens formed a biofilm in the control situation in the absence of any HelS1 EA extract. The anti-biofilm action was maximum against *B*. *cereus*, *S*. *epidermidis*, and *S*. *dysenteriae* followed by other pathogens with an inhibition percentage of 79.08 to 93.09 (Supplementary Figure 3).

### Ethyl Acetate Fraction of HelS1 Blocks the Energy Metabolism of the Pathogens

**FIGURE 4:**
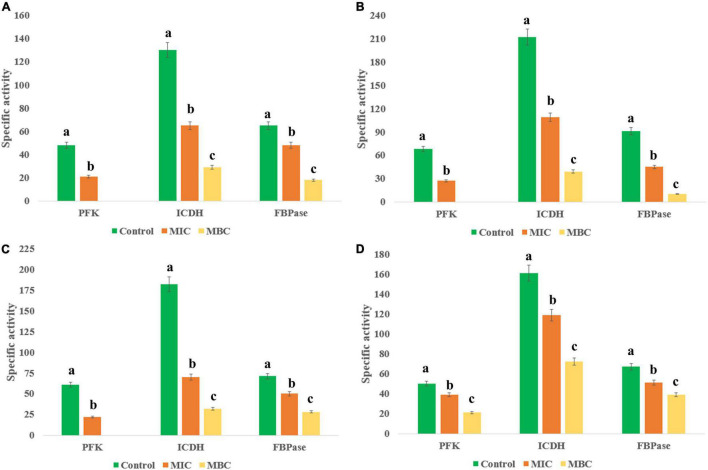
Effect of different concentrations of HelS1 ethyl acetate (EA) extract on the essential enzymes of microorganisms (**A** MRSA, **B** *L. monocytogenes*, **C** *V. parahaemolyticus*, and **D** *P. aeruginosa*) involved in central energy (carbohydrate) metabolism. Values on the graphs are the means ± standard error (SE) of the three replicates. Tukey’s multiple comparison test was performed. The different letters a, b, and c in each case [Control, MIC, minimum bactericidal concentrations (MBC)] represent a significant difference between them (At, *p* < 0.05).

The EA fraction of HelS1 directly blocks the actions of necessary enzymes (PFK, ICDH, and FBPase) involved in the central carbohydrate metabolism of bacterial pathogens. Gram-positive pathogens face the worst effect in comparison to the Gram-negative ones. At lethal doses (MBC), the pathogens face a severe stress situation (total blockage in energy metabolism), leading to a drastic reduction in enzymatic activity (Figure 4).

### Synergistic Activity of Ethyl Acetate Fraction of HelS1 With Standard Antibiotic

Commercially available antibiotic ciprofloxacin, in combination with EA fraction of HelS1 in different concentrations, was tested for synergistic antibacterial action against MRSA. A checker-board study elucidated that the combination of 6.25 μg ml^–1^ of EA fraction and 0.3 μg ml^–1^ of ciprofloxacin have synergistic activity against MRSA with ∑FIC of 0.425. The other higher combinations of 12.5 μg ml^–1^ (EA fraction) and 0.3 μg ml^–1^ (antibiotic-ciprofloxacin), 25 μg ml^–1^ (EA fraction), and 0.2 μg ml^–1^ (ciprofloxacin) were found to be synergistic too. No combinations were found to be antagonistic in this case. Different OD values in the checkerboard represent the growth of MRSA in different combinations of the two interacting agents (Table 2).

**TABLE 2:** Checkerboard-based representation of the cumulative effect of ethyl acetate (EA) fraction of HelS1 and antibiotic ciprofloxacin on the growth of methicillin-resistant *Staphylococcus aureus* (MRSA).

Ciprofloxacin (μgmL^–1^)	0.9	0.009	0	0	0	0	0	0
	0.7	0.059	0	0	0	0	0	0
	0.5	0.101	0	0	0	0	0	0
	0.3	0.4	0.32	0	0	0	0	0
	0.2	0.541	0.496	0.374	0.358	0	0	0
	0.1	0.689	0.598	0.402	0.376	0.296	0.198	0
	0	0.729	0.601	0.5	0.409	0.319	0.207	0
		0	3.125	6.25	12.5	25	50	100
	**EA extract of HELS1 (μgmL^–1^)**							

### Optimisation of the Culture Conditions for the Enhancement of Antibacterial Productions

OVAT optimisation revealed that endophytic HelS1 exhibits maximum antibacterial action when grown on fructose concentration (a carbon source)-7 gm L^–1^, peptone concentration (a nitrogen source)-4 g L^–1^, MpH of 6.8 and fermentation time of 8 days, NaCl concentration, 0.5 g L^–1^ (Supplementary Table 3). After the OVAT optimisation, RSM technique was applied using a three-level BBD, which involved the most important four factors-peptone concentration, fructose concentration, MpH, and fermentation time to elucidate the optimum antibacterial production by the isolate. The statistical model predicted the maximum antibacterial production at five replicated center points (Supplementary Table 4). The following equation was suggested by the statistical design, which provides maximum antibacterial production with minimum utilisation of necessary nutrients. YZOI = 5.3389–1.2915x_1_–1.3967x_2_–1.0901x_3_–1.3714x_4_–1.2567x_1_x_2_–1.3698x_1_x_3_ +1.1459x_1_x_4_–1.3789x_2_x_3_ + 1.3273x_2_x_4_–1.1336x_3_x_4_–1.6566x_1_^2^–1.6395x_2_^2^–1.5041x_3_^2^–1.8542x_4_^2^. x_1_, x_2_, x_3_, and x_4_ are the coded factors of fructose concentration, peptone concentration, medium-pH, and fermentation time, respectively. The goodness of fit of RSM and experimental outputs were evaluated by regression analysis (Supplementary Table 5). Contour and 3D plots were created by Minitab to focus on the different variables for the optimum antibacterial action (Supplementary Figure 4). It was found that there was an ignorable marginal deviation between the observed 22.33 ± 0.58 (mm) and predicted 22.66 ± 0.58 (mm) antibacterial response, following the model’s proposed fermentation conditions - fructose concentrations (6.868 g L^–1^), peptone concentrations (3.791 g L^–1^), M-pH-6.75, and fermentation time of 191.5 h.

### Antifungal Activity of HelS1 Volatile Organic Compounds

**FIGURE 5:**
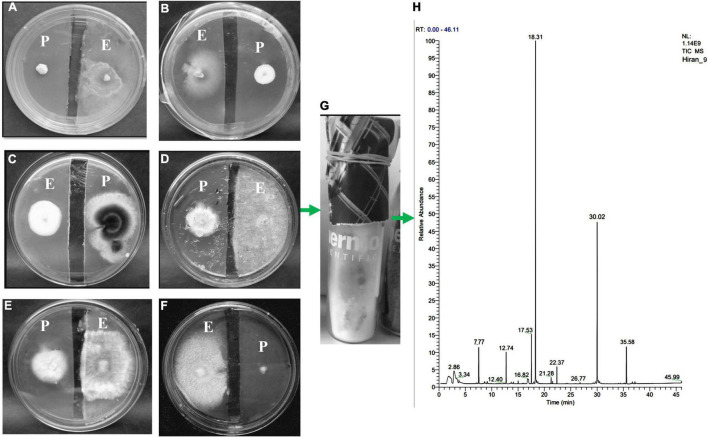
An antifungal gas test performed by a split plate method **(A–F)**. The growth of pathogenic [**(A)** *Geotrichum candidum*, **(B)** *Botrytis cinerea*, **(C)** *Cercospora beticola*, **(D)** *Rhizoctonia solani*, **(E)** *Pythium ultimum*, **(F)** *Cercospora beticola*] fungi has been inhibited by the VOCs of endophytic fungi HelS1 (except in one case-C). **(G)** Represents how endophytic fungi are grown on GC-glass vials for VOC analysis. **(H)** GC-chromatogram of the VOCs produced by the endophytic HelS1.

Volatile Organic Compounds of HelS1 exhibited anti-fungal action against a variety of pathogenic fungi from broad taxonomic groups like ascomycetes, basidiomycetes, and oomycetes (Table 3). HelS1 derived-VOCs expressed varying degrees of inhibition in plate assays against eight fungal pathogens. Maximum inhibitory effect (varied between 31.9 and 70.9%) of the VOCs was detected after 7 days of growth of HelS1 endophytic fungi (Figure 5). Out of eight test fungi, *F*. *oxysporum* (70.9%) and *G*. *candidum* (67.1%) were found to be maximally inhibited by the VOCs. Other pathogens (*P*. *ultimum*, 61.1%; *B*. *cinerea*, 54.9%; *R*. *solani*, 51.1%) were also inhibited in a moderate range. There was no inhibition for *C*. *beticola*, and *C*. *ulmi*, and minimum inhibition against *A*. *fumigatus* and *A*. *alternata*. VOCs of HelS1 are found to be biologically selective.

**TABLE 3:** Anti-fungal activity of an 8-day old endophytic fungus against selected fungal pathogens.

Fungal pathogens	Inhibition (%) after 168 h of treatment*^a^*	IC_50_ of artificial atmosphere after 72 h of treatment (μL 50 mL^–1^)	IC_50_ as μL mL^–1^ of air space needed for 50% inhibition^b^
*Geotrichum candidum*	67.1 ± 0.8	13.8 ± 0.7	0.276
*Botrytis cinerea*	54.9 ± 1.0	22.8 ± 0.5	0.456
*Cercospora beticola*	No inhibition	No inhibition	–
*Rhizoctonia solani*	51.1 ± 0.7	21.8 ± 0	0.436
*Pythium ultimum*	61.1 ± 0.9	13.6 ± 0	0.272
*Fusarium oxysporum*	70.9 ± 1.9	11.1 ± 0.1	0.222
*Aspergillus fumigatus*	33.39 ± 0.7	25.2 ± 0.6	0.504
*Alternaria alternata*	31.9 ± 1	29.2 ± 0.9	0.584
*Ceratocystis ulmi*	No inhibition	36.0 ± 1	0.72

*^a^Antifungal action was calculated as a percentage of pathogenic fungal growth inhibition relative to the growth of the same pathogenic fungi under control conditions.*

*^b^Concentration of VOCs’ artificial mixture (prepared with standards) in microlitres per millilitre of air space required to produce a 50% reduction of the pathogenic test fungi.*

### Effect of Substrate on Volatile Organic Compounds Production

Endophytic HelS1 was grown on several solid media (with changing nitrogen and carbon sources) to check the maximum production of bioactive volatiles. The VOCs were of characteristic odour due to maximum occurrence of naphthalene and azulene compounds, which smell like mothball. The increase in relative amounts of naphthalene and azulene emits a higher frequency of mothball-like odour, which is tested by five different lab-associated persons with a different olfactory score of 0–5. Finally, the most suitable media with a high olfactory score were designated as the most suitable VOC-emitting solid media for the selected isolate HelS1 (Table 4). Modified PDA (instantly smashed potato, 4 g L^–1^; yeast extract; urea, 0.2 g L^–1^; and dextrose, 40 g L^–1^) with necessary salts was found to be the most suitable media composition for maximum VOCs emission.

**TABLE 4:** Effect of medium compositions on VOCs production by endophytic fungi with qualitative olfactory observations obtained from independent ratings of 0–5, 5 being the maximum.

Medium composition	Independent olfactory score (0–5)
Beef extract	1
Tryptone	1.5
Peptone	0
Yeast extract	1.5
Yeast extract + Cellulose	2.5
Tryptone + Cellulose	2.5
Yeast extract+ Dextrose	3
Tryptone + Dextrose	3
Yeast extract + Malt extract	2.5
Tryptone + Malt extract	2.0
PDA	3
CDA	3.5
Oatmeal	3
Instantly smashed Potato +dextrose agar	
Modified PDA	5
Modified CDA	4
Modified Oatmeal	3

### Evaluation of IC_50_ Value of the Artificial Volatile Organic Compounds Mixture

Volatile organic compounds emitted by endophytic HelS1 were detected, and some of those selected chemicals were mixed in a proper ratio (as emitted by the endophyte and recorded by the GC-MS). Chemicals (whose standards were available) like 4-thujanol, naphthalene, 1,2-ethyl isobutyrate, undecane, azulene, and propionic acid were mixed in an exact ratio, and IC_50_ (in the complete air space of the test Petri plate) value of that artificial mixture was determined. The IC_50_ value was calculated, which ranged between 11.1 μg ml^–1^ and 36 μg ml^–1^ for 50% inhibition of the pathogenic fungal growth (Table 3). There was complete inhibition of fungal growth (except *C*. *beticola*) when 65 μL of the artificial mixture was introduced. The requirement of an artificial mixture for 50% inhibition of mycelial growth varied from organism to organism. Growth of *G*. *candidum*, *P*. *ultimum*, and *F*. *oxysporum* was found to be 50% ceased at a concentration of 13.8, 13.6, and 11.1 μL, respectively, which represents the high sensitivity of the test pathogens toward the artificial mixture. Likewise, these three pathogens were also maximally sensitive to VOCs of HelS1. Another test fungi (*R*. *solani*, *B*. *cinerea*, and *A*. *fumigatus*) with moderate IC_50_ values of 21.8, 22.8, and 25.2 μL also exhibited moderate sensitivity to the VOCs of HelS1. But, in the case of *C*. *ulmi*, there was 50% inhibition of mycelial growth at 36 μL of artificial mixture, whereas the fungal VOCs were unable to inhibit its growth. Again, in the case of *A*. *alternata*, the fungal VOCs were found to be more efficient in comparison to the artificial mixture in terms of growth inhibition (high IC_50_ value). The fungal VOCs, as well as the artificial mixture, were ineffective against *C*. *beticola*.

### Thin Layer Chromatographic Analysis and Antibacterial Action of the Active Fractions

**FIGURE 6:**
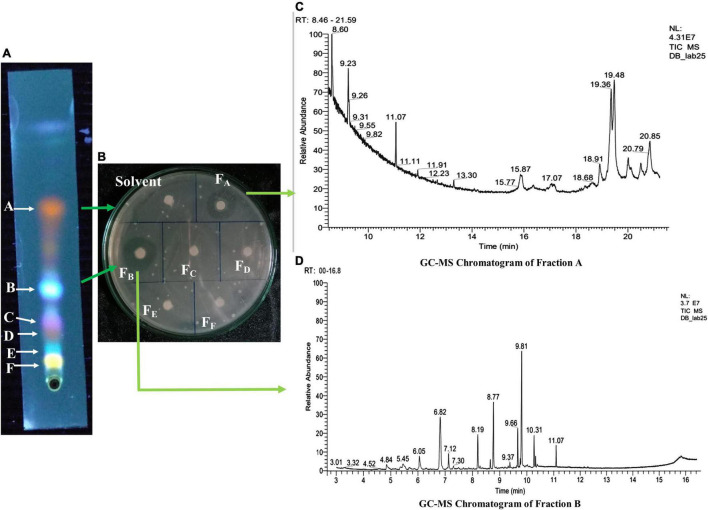
**(A)** Thin-layer chromatographic (TLC) analysis of EA fraction of HelS1. **(B)** Zones of inhibition produced by bioactive compounds present in fractions (F_*A*_–F_*F*_) obtained from a TLC plate against pathogenic bacteria-MRSA. **(C)** GC-MS chromatogram of Fraction A showing the peaks of bioactive compounds. **(D)** GC-MS chromatogram of Fraction B, showing the peaks of bioactive compounds.

Ethyl acetate fraction of HelS1 revealed six different bands under UV light (366 nm) after TLC analysis (Figure 6). Each fraction from six different bands was tested for antibacterial action, and bands A and B with an Rf value of 0.68 and 0.4, respectively, expressed antibacterial action against MRSA with a clear zone of inhibition of 17 and 21 mm.

### Detection of Bioactive Components

Bioactive compounds present on both the bands (A and B) of TLC with antibacterial activity were identified by the GC-MS-NIST library (Figure 6). Tables 5, 6 represent all the compounds along with their respective RT (retention time), area percentage, quality score, MW (molecular weight), and chemical formula. The chemical structures of the bioactive compounds along with their mass spectra are represented in Supplementary Figure 5. The compounds present in the band A are 1-H-indene 1 methanol acetate (26.44% with an RT of 9.81 min and a quality score of 96), tetroquinone (15.29% with an RT of 8.77 min and a quality score of 91.29), eucalanone (12.39% with an RT of 6.82 min and a quality score of 95.08), etc. Other compounds are methyl benzoate, ethyl benzoate, 3-butyn-1-ol, cholan-16-one, and ethanol (Table 4).

**TABLE 5:** A list of bioactive compounds present on the Spot A of the TLC plate identified by GC-MS analysis.

RT (min)	Total area (%)	Possible compounds	MW (g mol^–1^)	Chemical formula	Quality
8.60	11.40	Cholan-16-on, 23-methyl*^a^*	358	C_25_H_42_O	91
9.23	17.11	1,2-benzenedicarboxylic acid diethyl ester*^a^*	222.24	C_12_H_14_O_4_	97
11.07	8.26	2,4-dimethylbenzaldehyde*^a^*	134	C_9_H_10_O	98
11.91	2.07	Ethoxysympathol di PFP*^a^*	487	C17H_15_F_10_NO_4_	99
13.30	3.30	Unknown*^b^*	293	C_20_H_37_O	53
15.87	4.13	Ethyl decanoate*^a^*	200	C_12_H_24_O_2_	90
18.91	3.72	Unknown*^b^*	282	C_19_H_38_O	41
19.36	17.11	*Trans* 1, 2-diethyl-trans-2-decalinol*^a^*	182	C_12_H_22_O	87
19.48	18.25	*N*, *N*-diphenyl-2-nitro-thio benzamide*^a^*	334	C_19_H_14_N_2_O_2_S	98
20.31	4.55	Unknown*^b^*	148	C_10_H_12_O	51
20.59	5.371	Unknown*^b^*	130	C_8_H_18_O	39
20.85	7.22	3 fluoro-5-[1-hydrox-2-(methyl amino) ethyl phenol]^ a^	185	C_9_H_12_FNO_2_	92

*Compounds detected in the control PDB liquid are not reported in the table.*

*^a^Compounds with a quality score ≥60 are identified with the spectral database of NIST.*

*^b^Compounds with a quality score less than 60 are considered as unknown.*

**TABLE 6:** A list of bioactive compounds present on the Spot B of the TLC plate identified by GC-MS analysis.

RT (min)	Total area (%)	Possible compounds	MW (g mol^–1^)	Chemical formula	Quality
4.84	2.07	Methyl benzoate*^a^*	136	C_8_H_8_O_2_	68.23
5.45	1.66	Unknown*^b^*	296	C_21_H_44_	53
6.05	3.72	Unknown*^b^*	308	C_22_H_44_	58
6.82	12.39	Eucalanone*^a^*	390	C_22_H_14_O_7_	89.79
7.12	4.13	Ethylbenzoate*^a^*	134	C_9_H_10_O_2_	95.08
7.30	0.83	3-butyn-1-ol*^a^*	63.79	C_4_H_6_O	71.87
8.19	8.27	Unknown*^b^*	138	C_8_H_10_O_2_	42
8.77	15.29	Tetroquinone*^a^*	172	C_6_H_4_O_6_	91.29
9.37	0.83	Unknown*^b^*	320	C_20_H_44_O_12_	43
9.66	9.50	Menthyl acetate*^a^*	198	C_12_H_22_O_2_	69.04
9.81	26.44	1-H-indene 1 methanol, acetate^ a^	188	C_12_H_12_O_2_	96
10.31	8.68	Cholan-16-one, 3-(2-hydroxypropoxy)-cyclic 1, 2-propanedyl acetal*^a^*	358	C_25_H_42_O	88
11.07	6.20	Ethanol*^a^*	46	C_2_H_5_OH	92

*Compounds detected in the control PDB liquid are not reported in the table.*

*^a^Compounds with a quality score ≥60 are identified with the spectral database of NIST.*

*^b^Compounds with a quality score of less than 60 are considered unknown.*

Band B contains *N*, *N*-diphenyl-2-nitro-thio benzamide (18.25% with an RT of 19.48 min and a quality score of 98), *Trans* 1, 2-diethyl-trans-2-decalinol (17.11% with an RT of 19.36 and a quality score of 87), 1,2-benzene dicarboxylic acid diethyl ester (17.11% with an RT of 9.23, with a quality score of 97), etc. Other compounds are 2, 4-dimethyl benzaldehyde, ethyl decanoate, ethoxysympathol di PFP, and cholan-6-on, 23-methyl- (Table 6).

The SPME analysis revealed the occurrence of volatile compounds like naphthalene (47% with an RT of 18.31 min), azulene (22.54% with an RT of 30.02 min), 4-thujanol (7.04% with an RT of 17.53 min), propionic acid (5.63% with an RT of 35.58 min), benzene, 1, 3 dimethyl (4.7% with an RT of 12.74 min) (Table 7). Other compounds are undecane, 1,2-ethyl iso-butyrate, etc (Figure 5).

**TABLE 7:** GC/MS- SPME analysis of the volatile organic compounds (VOCs) emitted by an 8-day-old endophytic *Curvularia eragrostidis* HelS1 grown on a modified PDA medium.

RT (min)	Total area (%)	Possible compounds	MW (g mol^–1^)	Chemical formula	Quality
2.86	2.35	Azulene-1,2-dicarboxylate*^b^*	214.17	C_12_H_6_O_4_	67
7.77	5.17	Diacetamide*^b^*	101.10	C_4_H_7_NO_2_	65
12.74	4.7	Benzene, 1, 3 dimethyl*^b^*	106.17	C_8_H_10_	63
16.82	0.43	Unknown*^c^*	296	C_21_H_44_	53
17.53	7.04	4-thujanol*^a^*	154.25	C_10_H_18_O	94
18.31	47	Naphthalene*^a^*	128.17	C_10_H_8_	100
21.28	0.94	Unknown*^c^*	136	C_8_H_8_O_2_	31
22.37	2.34	1,2-Ethyl iso-butyrate*^a^*	116.16	C_6_H_12_0_2_	91
26.77	0.94	Undecane*^a^*	156.31	C_11_H_24_	96
30.02	22.54	Azulene*^a^*	128.17	C_10_H_8_	100
35.58	5.63	Propionic acid*^a^*	74.08	CH_3_CH_2_COOH	92

*Compounds detected in the control PDA vial are not reported in the table.*

*^a^Denotes that the standard of those components was analysed, and mass spectra and retention time matched closely or completely with the respective fungal volatiles.*

*^b^Denotes that the components were identified according to the NIST library with a quality score of 60 or above, and no standards were used for comparison.*

*^c^Denotes that the components represent a quality score value less than 60.*

## Discussion

### Antibacterial Activity of *Curvularia eragrostidis* HelS1 Metabolites Against Human Pathogenic Bacteria

In the modern world, antimicrobial resistance is one of the prime problems that need efficient handling strategies. The conventional antibiotics (like aminoglycosides, chloramphenicol, fluoroquinolones, macrolides, and tetracycline) are losing their efficacy; organisms are evolving as multi-drug-resistant strains, and it is high time to switch to something new and novel to tackle the situation (Ness, 2010; Villavicencio et al., 2021). Natural products from the plant, fungal, and bacterial secondary metabolites are the most prominent candidate in this respect. Here, our focus is especially on endophytic fungal metabolites. We have selected the ethnomedicinal plant *Helicteres isora* as the source of endophytic fungi due to its immense utilisation as an anti-microbial and anti-oxidative agent amongst the local ethnic tribes of Jharkhand, India (Kumar and Singh, 2014). Presently, we have isolated eight endophytic fungi from the leaf and stem tissues of the explant. The most potent antibacterial isolate is a true endophyte as it is sterile even after inoculation in a leaf carnation medium, and, also, there is no occurrence of disease symptoms when the isolate is re-inoculated onto leaf tissues of the healthy *H*. *isora* plant (following the methods of Koch’s Postulate). The antibacterial activity of the mycelial EA extract of HelS1 is found to be lethal or bactericidal against six Gram-positive and seven Gram-negative human pathogens with MIC and MBC values of 12.5–400 μg ml^–1^ and 25–800 μg ml^–1^, respectively. Maximum antibacterial action (in terms of lower MIC, MBC, leakage of macromolecules, inhibition of energy metabolism) is exhibited against Gram-positive pathogens than the Gram-negative ones. Gram-negative ones possess two protecting structures – thick outer membrane and periplasmic space, which makes it very difficult for the antibacterial principles to penetrate, whereas Gram-positive cells with only an outer peptidoglycan layer have a higher susceptibility toward antibacterial components (Duffy and Power, 2001). The enhanced permeability of the bacterial cell membrane can be achieved as a result of the insertion of fungal metabolites, which can restrict cell growth, cause leakage of vital cellular metabolites, and can eventually lead to cell death (Shin et al., 2007).

The extracted antibacterial metabolites are non-proteinaceous and thermostable, which makes them suitable for further *in vivo* experimentation. EA is selected as the most suitable extraction agent in comparison to other solvents (ethyl ether, petroleum ether, and *n*-hexane) as the EA fraction showed the highest antibacterial action. Non-proteinaceous nature and thermostability of antibacterial components obtained from endophytic fungi are also reported by Santra et al. (2022). Malhadas et al. (2017) also reported the anti-bacterial action of the EA fraction of secondary metabolites of olive tree endophytes.

Our prime outcome of the study is an anti-MRSA activity of the fungal metabolites. MRSA is known to be a very fatal pathogen and causes a variety of disease in immune-compromised or healthy individuals (Zajmi et al., 2015). Extensive use of antibiotics and nosocomial infections has led to the development of such fatal strains (Doggrell, 2005). Anti-MRSA action of the EA fraction is further confirmed by the remarkable decrease in the number of CFUs paralleled to the higher increasing concentration of the anti-bacterial metabolites (as per the results of killing kinetics experiments).

The fungal metabolites disrupted the bacterial cell walls as there was leakage of intracellular macromolecules (DNA, protein, and K^+^ ions) into the extracellular environment. So, the metabolites exhibit a cidal mode of action. A similar type of an outcome is also reported by the endophytic metabolites of *A*. *alternata* against bacterial pathogens (Chatterjee et al., 2019, 2020).

Time kill curves are the most reliable way to detect the bactericidal or bacteriostatic nature of the different concentrations of antibacterial principles (Guidelines, 2006; CLSI, 2017). This curve elucidates the kinetics of bacterial killing *in vitro* and, on the other hand, also provides us with qualitative and quantitative data on antibacterial principles for further investigations. Zajmi et al. (2015) detected the anti-MRSA and anti-Staphylococcal action of artonin by determining MIC and MBC values from the time-kill curve.

The necessary enzymes involved in central carbohydrate metabolism are also affected by the fungal metabolites, and there was a sharp inhibition of those enzymes involved in energy metabolism upon the increase of a treatment dose, i.e., MIC and MBC. A similar type of finding was made by Santra et al. (2022) and Chatterjee et al. (2019) where endophytic fungal metabolites inhibit the three main enzymes of potent bacterial pathogens involved in carbohydrate metabolism.

Development of antibiotic resistance and formation of a biofilm are the two main key features of methicillin-resistant *S*. *aureus* (MRSA) infections in both health care (infects implanted devices like urinary catheters, prosthetics, contact lens, and cause skin, heart valve, bone, soft tissue infections, pneumonia, septicemia) and community settings (Gordon and Lowy, 2008). More than 80% of human bacterial diseases are due to biofilm-forming bacteria (Davies, 2003). Biofilms are the aggregation of bacterial populations shielded in a matrix of exopolysaccharides, which provide the bacterial cells, enhanced resistance to antibiotic treatments, and it has been found that biofilm-incorporated cells are more virulent than the individual cells (Akbari-Ayezloy et al., 2017; Piechota et al., 2018). Most of the ESKAPE (*Eetrococcus faecium*, *S*. *aureus*, *K*. *pneumoniae*, *Acinetobacter baumannii*, *Pseudomonas aeruginosa*, species of Enterobacter) pathogens are reported to develop a biofilm, which makes it very difficult to treat the infections (Mulani et al., 2019). Here, we have assessed the biofilm inhibitory action of fungal metabolites against three of the ESKAPE pathogens, and antibacterial metabolites of HelS1 successfully inhibited the biofilm formation (up to 79%) of those selected pathogenic bacteria.

The antimicrobial resistance can be, to some extent, tackled by administering excessively increased doses of antibiotics but that has some side effects and further worsens the situation. So, we have evaluated the synergistic action of antibiotics along with fungal metabolites by the checkerboard method, which elucidates the effective concentration of both the antibiotic and fungal metabolites at which maximum reduction in bacterial number takes place. It has been revealed that commercially available ciprofloxacin can inhibit bacterial growth at concentrations lower than its MIC when applied with HelS1 fungal metabolites. The cell wall-bursting action of antibacterial components promotes the better penetration of antibiotic ciprofloxacin to the pathogenic cells and the co-administration of both the drugs at specific concentrations (lower than their individual MIC values) can be an efficient disease management strategy. Co-administration of anti-fungal drug fluconazole and endophytic fungal metabolites also revealed the same result against *C*. *albicans* pathogenic cells (Chatterjee et al., 2019).

It is very necessary to keep any industrial-fermentation-based production line in a cost-efficient way, and optimisation of necessary growth parameters for the maximum production of the microbial product remains the primary target (Santra and Banerjee, 2021). To meet that criterion, we have optimised the fermentation parameters by the OVAT (one variable at a time) method coupled with RSM (response surface methodology) and BBD (Box Behnken Design). Additional carbon and nitrogen sources, medium pH, and fermentation time are optimised in this study, and the model equation is found to be statistically valid (confirmed by regression analysis), as well as fruitful in fixing the optimum parameters on a trial-and-error basis. The large F value of 1472.53 indicates the model’s significance. The higher value of *R*^2^ adj (99.01%) confirms the high degree of correlation between the experimentally measured and statistically predicted data. A very low lack of fit *F* value of 11.67 indicated that the model has a very negligible amount of error. The model *p* < 0.0001 states that the model is appropriate for the evaluation of antibacterial production. The system accuracy was confirmed by the lack of fit *P*-value, which is 0.297 (higher than 0.05). The linear and quadratic effects of fructose concentration, peptone concentration, fermentation time, and medium pH were significant (*p* < 0.0001) in this model. Finally, the model predicted the most efficient antibacterial action of a 22.66 ± 0.58-mm clear zone of inhibition at FC, 6.868 g L^–1^; PC, 3.791 g L^–1^; MpH, 6.75; FT, 191 h; and 30 min, which suits with our experimental outcome of a 22.33- ± -0.58-mm clear zone of inhibition.

OVAT and RSM techniques were also adopted by several workers for the production of the antibacterial compound, and bioactive exopolysaccharides from endophytic fungal (*Fusarium* sp. SD5, *Pestalotiopsis* sp. BC55, *Colletotrichum alatae* LCS1, *Cochliobolus* sp. APS1) isolates (Mahapatra and Banerjee, 2013, 2016; Santra and Banerjee, 2022a,b; Santra et al., 2022).

The bioactive compounds were first separated using TLC, and then the most effective two bands were analysed for their composition. GC-MS-based identification revealed the occurrence of 12 antibacterial compounds in Fraction A (ethyl decanoate, *N, N*-diphenyl-2-nitro-thio benzamide, trans 1, 2-diethyl-trans-2-decalinol, etc.) and 13 bioactive compounds in Fraction B (methyl benzoate, ethanol, menthyl acetate, 3-butyn-1-ol, etc.) out of which four were unknown without any match of previously known compounds. Most of the components have not been previously reported from any endophytic fungi and are new reports from the present study.

An artificial mixture was prepared using the compounds available in the form of a standard (from Sigma), which mimicked the compositions of Bands A and B. All the components were not available, and the artificial mixture contained some selected available components in an appropriate ratio as produced by the endophyte. Finally, the antibacterial action of the artificial mixture was found to be inferior to the natural endophytic extract, and it could be concluded from the result that endophytic metabolites are genuinely unique and represent a wide array of unknown/new or novel bioactive metabolites that need further attention for broad-spectrum industrial and commercial exploitation.

### Volatile Organic Compound-Mediated Anti-fungal Action

Volatile organic compounds represent a large group of diverse chemicals that possess a wide spectrum of biological activity (Santra and Banerjee, 2020b). Endophytic VOCs have been previously reported to be broad-spectrum antimicrobial in nature and also possess fuel potency (Strobel et al., 2010; Strobel et al., 2011). Endophytic *Muscodor albus* and several other species of Muscodor were isolated from different parts of the world along with *Phoma* sp., *Phomopsis* sp. and *Hypoxylon* sp., *Myrothecium innundatum*, which produces volatile antimicrobial and restricts the growth of severe phytopathogens (Daisy et al., 2002; Strobel, 2006; Mitchell et al., 2009; Banerjee et al., 2010a,b, 2014; Tomsheck et al., 2010; Saxena and Strobel, 2021). The present study also reports the anti-fungal action of bioactive VOCs emitted by the endophytic fungal isolate HelS1. The VOCs inhibit the growth of seven fungal pathogens, and the composition of VOCs has been identified using GC-MS. In total, nine compounds are detected in the HelS1-emitted VOC. The prime component was naphthalene, followed by azulene and 4-thujanol. Each of the components has its characteristic odour, but naphthalene being the most dominant one masks the other components’ smell. So, the overall smell of the fungal VOCs largely mimics the mothball like a strong pungent odour masking the other smell. 4-thujanol (also known as sabinene hydrate) is a natural bicyclic monoterpene with the smell of spiciness of black pepper detected in the VOCs. Azulene (an isomer of naphthalene) is present as the second major component and bears a similar smell to naphthalene. Ethyl iso-butyrate with fruity aromatic odour and another bioactive compound undecane with faint odour is also produced by the isolate. Organic acid-propionic acid with a pungent, rancid unpleasant odour is emitted in minute amounts by the isolate. Earlier, naphthalene and sabinene monohydrates were reported by Daisy et al. (2002) and Singh et al. (2011) from *Muscodor vitigenus* and *Phomopsis* sp., respectively. The endophytic VOCs are majorly found to be bio-active, and our experimental outcomes support the same (Banerjee et al., 2010a,b; Kudalkar et al., 2012; Mao et al., 2019; Pena et al., 2019).

The maximum production of the major compound naphthalene is detected at a medium composed of PDA, which is supplemented with yeast extract and urea (0.2 g L^–1^), dextrose (40 g L^–1^). The maximum production of mothball-like odour is confirmed by the individual olfactory score of lab members. An artificial mixture is prepared, following the ratio of components present in the fungal VOC. The IC_50_ value of the artificial mixture against 9 phytopathogens ranged from 13.6 ± 0 μL 50 mL^–1^ to 36. ± 1 μL 50 ml^–1^. Fungal pathogen *C*. *ulmi* is not at all inhibited by the endophytic VOCs but is inhibited with an IC_50_ value of 36 μL 50 ml^–1^ by the artificial mixture. In the case of *C*. *beticola*, both the fungal VOC and artificial mixture are unable to restrict its growth. In the case of fungal pathogens *F*. *oxysporum*, *G*. *candidum*, and *P*. *ultimum*, the maximum inhibition is found upon fungal VOC treatment, whereas the artificial mixture is most effective against *F*. *oxysporum*, *P*. *ultimum*, and *G*. *candidum*, respectively. So, there is a slight change in the mode of action between the fungal VOC and artificial mixture.

The findings suggest that, although, to some extent, the mimicry of the artificial mixture may yield similar results, but not in all the cases. It also elucidates that the presence of other compounds (either unknown or the not-detected ones) in the original natural VOC mixture may trigger the fungal growth inhibition in some cases, but not for all. Similar types of outcomes were also reported by VOCs of endophytic *Phomopsis* sp. isolated from *Odontoglossum* sp. (Singh et al., 2011). Medicinal plants always serve as the pool of diverse endophytic fungi, and our present outcome also supports the same (Gond et al., 2012; Dhayanithy et al., 2019). *H*. *isora* leaf harbours eight different types of endophytic fungi, and the biodiversity indices declare the significant assemblage of endophytes, colonising the leaf tissues of the plant. Endophytes of medicinal plants regularly represent unique volatile with multidomain bioactivity (Banerjee et al., 2010a,b).

The antifungal activity of these endophytic volatiles opens up new scopes for sustainable agricultural practices and deep ecological approaches where the myco-fumigation technique can be applied to restrict the growth of phytopathogens in living stock feeds and in post-harvest disease management. In the recent past, use of endophytic fungal volatiles in the management of post-harvest decay has gained interest, and our present investigational outcome is just a new addition to that list (Saxena and Strobel, 2021).

The test organisms used in this study are a potential causal agent of several dreadful diseases and devastate the agro-economy. *G*. *candidum* causes soft root rot in *Ipomea batatas*, and *B*. *cinerea* (also called grey mold) causes necrotrophic lesions in over 200 plant species but, predominantly, in wine grapes (Holmes and Clark, 2002; Williamson et al., 2007). *R*. *solani* causes damping-off in a wide range of hosts, especially in rapeseed and wheat, whereas *P*. *ultimum* is the causal agent of root rot and damping-off in 100s of crops, especially corn, wheat, fir, soybean, and potato (Verma, 1996; Agrios, 2005). The other two pathogens *F*. *oxysporum* and *A*. *alternata* are known to cause fusarium wilt, foot, and root rot in hundreds of crops, and brown and black leaf spots in tomato, tangerine, tobacco, and strawberry, respectively (Michielse and Rep, 2009; Meena et al., 2017). Another human pathogen *A*. *fumigatus*, a causal agent of aspergillosis in immune-compromised patients, is also inhibited by the fungal VOCs (Dagenais and Keller, 2009). Millions of dollars are invested, and harmful-toxic chemical formulations are used on a large scale to control these pathogens (Aktar et al., 2009; Zubrod et al., 2019). So, instead of using xenobiotic antifungals with severe side effects, the fungal isolate can be used as a potential biocontrol agent to have a safe, green, and sustainable environment. It is the first investigation of VOCs emitted by an endophytic *Curvularia eragrostidis* HelS1 isolated from the medicinally valuable *Helicteres isora* plant from the forests of East India.

## Conclusion

In a nutshell, endophytes are the all-square bioactive entities, and their volatile, non-volatile metabolites possess unique multi-domain bio-activity. Here, endophytic *Curvualria eragrostidis* HelS1 is found to be an effective anti-microbial producer. VOCs emitted by the isolate restrict the growth of dreadful phytopathogens and can be utilised as a tool for sustainable agriculture. The endophyte *C*. *eragrostidis* HelS1 can be commercialised as a myco-fumigator. This will be an alternative to synthetic anti-fungal and will act as a biocontrol agent in managing post-harvest diseases. In a world of dreadful diseases, it is the correct time to switch to the novel, non-toxic bio-metabolites, which will further support the concept of deep ecological movements. Not only in the arena of agriculture but also the isolate *Curvularia eragrostidis* HelS1 contributes to the field of pharmaceutical sciences where the bioactive metabolites can be a potent alternative to conventional antibiotics and may efficiently check the fatal diseases caused by multidrug-resistant Gram-positive and Gram-negative bacterial pathogens in the human population. Our outcomes open up rays of hope in the domain of novel, non-conventional bioactive product isolation from untapped biological sources, and endophytes from ethnomedicinal plants are found to be the major source in this respect.

## Data Availability Statement

The original contributions presented in this study are included in the article/Supplementary Material, further inquiries can be directed to the corresponding author.

## Author Contributions

HS and DB designed the research plan and prepared the manuscript. HS performed all the experiments and analysed the data statistically. DB coordinated the whole work and discussed the results. Both authors read the manuscript and approved it for submission.

## Conflict of Interest

The authors declare that the research was conducted in the absence of any commercial or financial relationships that could be construed as a potential conflict of interest.

## Publisher’s Note

All claims expressed in this article are solely those of the authors and do not necessarily represent those of their affiliated organizations, or those of the publisher, the editors and the reviewers. Any product that may be evaluated in this article, or claim that may be made by its manufacturer, is not guaranteed or endorsed by the publisher.
