# Supplementary material for: Broad-Spectrum Antimicrobial Action of Cell-Free Culture Extracts and Volatile Organic Compounds Produced by Endophytic Fungi Curvularia Eragrostidis
Source: Front Microbiol. 2022 Jun 23;13:920561. doi: 10.3389/fmicb.2022.920561 (PMC9260591; doi:10.3389/fmicb.2022.920561)
Supplement: Supplementary file 1 [file Data_Sheet_1.doc]

**Materials and method for the detection of colonisation frequency and diversity indices of endophytic fungi isolated from leaf tissues of *H*. *isora***

The colonisation frequency of the endophytic fungal isolates was determined using the protocols of Hata and Futai (1995). %CF= (N_col_/N_t_) X 100, where N_col_ = number of segments of plant tissue colonized by each fungus and N_t_= total number of segments of plant tissue studied. The following diversity indices were further evaluated- Simpson’s index of dominance (D) was determined by the following formula (Simpson, 1951). D=Σ (n/N)^2^, where n= the total number of isolates of a particular species, while N= the total number of isolates of all species. Simpson’s diversity index= 1-D. Species richness= S/√N, where S= total number of species. The Shannon-Wiener index (H’) was estimated by Σ (pi Ln pi), where pi=n/N. Species evenness E was also evaluated by H’/ln S.

**Supplementary Table 1** Colonisation frequency of endophytic fungi isolated from leaf tissues of *Helecteris isora*.

| **Name of the endophytes** | **No. of isolates** | **CF (Colonisation frequency)** |
| --- | --- | --- |
| *Fusarium* sp. 1 | 14 | 9.33 |
| *Fusarium* sp. 2 | 11 | 7.33 |
| *Pestalotiopsis* sp. | 19 | 12.67 |
| *Penicillium* sp. | 9 | 6.66 |
| *Aspergillus* sp. 1 | 10 | 6 |
| *Aspergillus* sp. 2 | 7 | 4.66 |
| Sterile isolate 1 (HelS1) | 21 | 14 |
| Sterile isolate 2 (HelS2) | 16 | 10.66 |

**Supplementary Table 2** Diversity indices of endophytic fungi isolated from the leaf tissues of the host plant.

| **Diversity indices** | **Respective values** |
| --- | --- |
| Simpson’s dominance | 0.137 |
| Simpson’s diversity | 0.863 |
| Species richness | 0.773 |
| Shanon-Wiener | 2.002 |
| Evenness | 0.962 |

**Supplementary Table 3** Effect of different physical conditions and chemical supplements on biomass and antibacterial activity (ZOI-zone of inhibition) of HelS1 against MRSA (Methicillin resistant *Staphylococcus aureus*).

| **Parameters** | **Effectors** | **Percentage added**  **(g/L)** | **Biomass (g/L)** | **Antibacterial activity (ZOI in**  **mm)** |
| --- | --- | --- | --- | --- |
| Incubation time (in days) | 4 | **-** | 5.701±0.125 | 6±0 |
|  | 6 | **-** | 5.343±0.045 | 7.33±0.58 |
|  | **8** | **-** | **5.909±0.029** | **10.67±0.58** |
|  | 10 | **-** | 4.985±0.078 | 7±1 |
| Incubation temperature (ᵒC) | 24 | **-** | 7.109±0.091 | 10.33±0.58 |
|  | **26** | **-** | **8.863±0.030** | **13.33±0.58** |
|  | 28 | **-** | 6.903±0.071 | 10.67±0.58 |
|  | 30 | **-** | 4.079±0.058 | 8±0 |
| Initial medium pH | 6 | **-** | 3.190±0.009 | 3.33±0.58 |
|  | 6.4 | **-** | 4.607±0.011 | 4.33±0.58 |
|  | **6.8** | **-** | **7.071±0.018** | **7±1** |
|  | 7.2 | **-** | 6.074±0.019 | 9±0 |
|  | 7.6 | **-** | 5.019±0.006 | 6.33±0.58 |
| Additional carbon source | Starch | 1 | 8.769±0.001 | 9.67±0.58 |
|  | Glucose | 1 | 8.079±0.019 | 8.67±0.58 |
|  | **Fructose** | **1** | **9.039±0.041** | **11±1** |
|  | Maltose | 1 | 8.019±0.011 | 9±1 |
| Additional nitrogen sources | Tryptone | 2 | 9.091±0.014 | 10.33±0.58 |
|  | **Peptone** | **2** | **9.39±0.019** | **13±1** |
|  | Yeast Extract | 2 | 9.089±0.009 | 9±0.58 |
| Fructose concentration | Fructose | 4 | 9.134±0.001 | 11.33±0.58 |
|  |  | 5 | 9.039±0.009 | 12±0 |
|  |  | **7** | **10.998±0.009** | **13.33±0.58** |
|  |  | 8 | 9.991±0.001 | 17±0 |
|  |  | 10 | 9.066±0.005 | 12.33±0.58 |
|  |  | 12 | 9.059±0.004 | 11.33±0.58 |
| Peptone concentration | 1 | - | 9.109±0.010 | 9.67±0.58 |
|  | 3 | - | 10.719±0.011 | 12.33±0.58 |
|  | **4** | **-** | **10.069±0.004** | **16.67±0.58** |
|  | 6 | - | 9.938±0.009 | 14.67±0.58 |
| Different metal ions | **NaCl** | **0.05** | **10.049±0.061** | **13±1** |
|  | KCl | 0.05 | 9.011±0.016 | 9.67±0.58 |
|  | MgCl2 | 0.05 | 6.809±0.068 | 10.33±0.58 |
|  | CaCl2 | 0.05 | 4.011±0.001 | 11.33±0.58 |
| NaCl concentration |  | 0.25 | 8.158±0.051 | 12.33±.0.58 |
|  |  | **0.5** | **10.097±0.098** | **15±0** |
|  |  | 1 | 9.009±0.008 | 14.67±0.58 |

Antibacterial action is tested using the endophytic culture extract at each fermentation condition and finally the combination at which better clear zone (represented as **bold** letters) was achieved was used for further investigation.

| **Independent variables** | | | | | **Response (ZOI-in mm)** | |
| --- | --- | --- | --- | --- | --- | --- |
| **Run** | **X_1_ (FC)** | **X_2_ (PC)** | **X_3_ (MpH)** | **X_4_ (FT)** | **Measured** | **Predicted** |
| 1 | -1 (6) | -1 (3) | 0 (6.8) | 0 (8) | 23.51 a_1_ | 24.62 a_1_ |
| 2 | 1 (8) | -1 | 0 | 0 | 22.61 a_2_ | 22.71 a_2_ |
| 3 | -1 | 1 (5) | 0 | 0 | 21.56 a_3_ | 21.17 a_3_ |
| 4 | 1 | 1 | 0 | 0 | 20.56 a_4_ | 19.16 a_4_ |
| 5 | 0 (7) | 0 (4) | -1 (6.4) | -1 (7) | 22.40 a_5_ | 21.85 a_5_ |
| 6 | 0 | 0 | 1 (7.2) | -1 | 22.43 a_6_ | 22.69 a_6_ |
| 7 | 0 | 0 | -1 | 1 (9) | 22.58 a_7_ | 22.02 a_7_ |
| 8 | 0 | 0 | 1 | 1 | 19.49 a_8_ | 19.72 a_8_ |
| 9 | -1 | 0 | 0 | -1 | 23.19 a_9_ | 23.02 a_9_ |
| 10 | 1 | 0 | 0 | -1 | 20.44 a_10_ | 20.29 a_10_ |
| 11 | -1 | 0 | 0 | 1 | 19.68 a_11_ | 20.85 a_11_ |
| 12 | 1 | 0 | 0 | 1 | 18.57 a_12_ | 19.67 a_12_ |
| 13 | 0 | -1 | -1 | 0 | 23.77 a_13_ | 24.70 a_13_ |
| 14 | 0 | 1 | -1 | 0 | 20.57 a_14_ | 21.03 a_14_ |
| 15 | 0 | -1 | 1 | 0 | 23.35 a_15_ | 23.86 a_15_ |
| 16 | 0 | 1 | 1 | 0 | 20.38 a_16_ | 20.47 a_16_ |
| 17 | -1 | 0 | -1 | 0 | 23.85 a_17_ | 23.13 a_17_ |
| 18 | 1 | 0 | -1 | 0 | 20.81 a_18_ | 21.16 a_18_ |
| 19 | -1 | 0 | 1 | 0 | 23.54 a_19_ | 20.40 a_19_ |
| 20 | 1 | 0 | 1 | 0 | 20.51 a_20_ | 20.45 a_20_ |
| 21 | 0 | -1 | 0 | -1 | 24.87 a_21_ | 24.06 a_21_ |
| 22 | 0 | 1 | 0 | -1 | 19.55 a_22_ | 20.73 a_22_ |
| 23 | 0 | -1 | 0 | 1 | 24.80 a_23_ | 22.84 a_23_ |
| 24 | 0 | 1 | 0 | 1 | 19.20 a_24_ | 19.16 a_24_ |
| 25 | 0 | 0 | 0 | 0 | 27.42 a_25_ | 27.45 a_25_ |
| 26 | 0 | 0 | 0 | 0 | 27.41 a_26_ | 27.45 a_26_ |
| 27 | 0 | 0 | 0 | 0 | 27.54 a_27_ | 27.45 a_27_ |
| 28 | 0 | 0 | 0 | 0 | 27.40 a_28_ | 27.45 a_28_ |
| 29 | 0 | 0 | 0 | 0 | 27.55 a_29_ | 27.45 a_29_ |

**Supplementary Table 4** Experimental design and results of the Box-Behnken design for the optimisation of the antibacterial activity of the fungal isolate HelS1

FC-Fructose concentration, PC-Peptone concentration, MpH-Medium pH, FT-fermentation time

There are 29 fermentation combinations where in each combination there are four fermentation media parameters (x_1_-x_4_). Each media parameters (FC-fructose concentration, PC-peptone concentration, MpH-medium pH, FT-fermentation time) are utilised in three separate concentrations (+1-just one value higher than the optimum, 0-the optimum condition and -1-one value lower than the optimum). Measured response is the one that has been calculated using the medium composition manually and predicted response is the statistically expected antibacterial action anticipated by the statistical model.

In each case there has been no difference among the measured and predicted response which indicates the accuracy of the statistical procedure.

One-way ANOVA (Tukey’s Multiple Comparison test) was performed to check the potential statistical differences (P<0.05) between the measured and predicted antibacterial action (zone of inhibition). There were no statistical differences between each data set (row wise) and similar letters (a_1_-a_29_) in each row indicates the data are same and lacks statistical differences.

**Supplementary Table 5** ANOVA for response surface quadratic regression model of antibacterial production by endophytic HelS1

| **Source** | **DF** | **Sum of Squares** | **Mean of Squares** | **F-Value** | **P-Value** |
| --- | --- | --- | --- | --- | --- |
| Model | 14 | 199.020 | 14.2157 | 1472.53 | <0.0001 |
| FC (x_1_) | 1 | 11.505 | 11.5052 | 2310.14 | <0.0001 |
| PC (x_2_) | 1 | 36.820 | 36.8200 | 1132.47 | <0.0001 |
| M pH (x_3_) | 1 | 1.562 | 1.5624 | 1111.38 | <0.0001 |
| FT (x_4_) | 1 | 5.880 | 5.8800 | 1025.18 | <0.0001 |
| FC*FC (x_1_^2^) | 1 | 63.947 | 63.9474 | 1166.38 | <0.0001 |
| PC*PC (x_2_^2^) | 1 | 37.318 | 37.3181 | 1152.90 | <0.0001 |
| M pH*M pH (x_3_^2^) | 1 | 41.432 | 41.4319 | 1146.53 | <0.0001 |
| FT*FT (x_4_^2^) | 1 | 73.059 | 73.0592 | 1194.42 | <0.0001 |
| FC*PC (x_1_x_2_) | 1 | 0.003 | 0.0025 | 1110.00 | <0.0001 |
| FC*M pH (x_1_x_3_) | 1 | 0.000 | 0.0001 | 1011.00 | <0.0001 |
| FC*FT (x_1_x_4_) | 1 | 0.601 | 0.6006 | 1101.53 | <0.0001 |
| PC*M pH (x_2_x_3_) | 1 | 0.013 | 0.0132 | 1301.01 | <0.0001 |
| PC*FT (x_2_x_4_) | 1 | 0.031 | 0.0306 | 1609.03 | <0.0001 |
| M pH*FT (x_3_x_4_) | 1 | 2.465 | 2.4649 | 1427.17 | <0.0001 |
| Error | 14 | 15.878 | 1.1341 |  |  |
| Lack-of-Fit | 10 | 15.860 | 1.5860 | 11.67 | 0.297 |
| Pure Error | 4 | 0.018 | 0.0044 |  |  |
| Total | 28 |  |  |  |  |
| R^2^ |  | 99.61% |  |  |  |
| R^2^ (adj) |  | 99.01% |  |  |  |
| R^2^ (pred) |  | 99.78% |  |  |  |

PC-peptone concentration, FC-fructose concentration, M-pH-medium pH, FT-fermentation time

Supplementary figure 1- Colonization frequency of endophytic fungal isolates of *H*. *isora*

Supplementary figure 2- Leakage of intracellular macromolecules into the extracellular environment A-DNA content B- K^+^ ions and C- protein content. Values on the graphs are the means ± Standard error (SE) of the three replicates. Tukey’s multiple comparison test was performed. The different letters a and b in each case (for each bacterial pathogen values at 6 and 24 hours) represents a significant difference between them (At, p<0.05).

Supplementary figure 3- Bio-film inhibition by HelS1 EA extract a- against MRSA. b- Change in OD values of the MRSA bio-film formation after treatment with different concentrations of the HelS1 EA extract. Values on the graphs are the means ± Standard error (SE) of the three replicates. Tukey’s multiple comparison test was performed. The different letters a, b, c, d, e, f indicates significance difference in comparison to control (At, p<0.05). Same letters indicate no difference.

Supplementary figure 4- The 3D plot with 2D projection and contour plot showing the most important interactions of factors in RSM optimization of antibacterial activity by HelS1. (A1, A2) between peptone concentration (PC) vs. fructose concentration (FC) at fermentation time (FT) 8 days and medium pH 6.8 (MpH); (B1, B2) between MpH (6.8) and FC (7gmL^-1^) at FT (8 days) and PC (4gmL^-1^); (C1, C2) between FT (8 days) and FC (7gmL^-1^) at PC (4gmL^-1^) and MpH (6.8); (D1, D2) between MpH (6.8) and PC (4gmL^-1^) at FT (8 days) and FC (7gmL^-1^); (E1, E2) between FT (8 days) and PC (4gmL^-1^) at MpH (6.8) and FC (7gmL^-1^); (F1, F2) between FT (8 days) and MpH (6.8) at PC (4gmL^-1^) and FC (7gmL^-1^).

Supplementary figure 5- Chemical structures of the VOCs produced by the endophyte along with their Mass spectra a- Napthalene, b-Tetroquinone, c- Eucalanone, d- 1H-Indene-1-methanol, acetate, e- Cholan-16-on, 23-methyl-, f- menthyl acetate, g- 3-methyl butyn 1-ol, h- Methyl benzoate, i- N, N-diphenyl-2-nitro-thio benzamide, j-2,4-Dimethylbenzaldehyde, k-Ethoxysympathol di PFP, l- Ethyl decanoate

Supplementary figure 1

Supplementary Figure 2

Supplementary Figure 3

Supplementary Figure 4

Supplementary Figure 5
